# The Shape and Function of Solid Fascias Depend on the Presence of Liquid Fascias

**DOI:** 10.7759/cureus.6939

**Published:** 2020-02-10

**Authors:** Bruno Bordoni

**Affiliations:** 1 Physical Medicine and Rehabilitation, Foundation Don Carlo Gnocchi, Milan, ITA

**Keywords:** fascia, myofascial, osteopathic, fascintegrity, skeletal muscle, physiotherapy

## Abstract

Scientific research is not a showcase of his own talent or own resources, it is a chance to improve common knowledge on certain topics for the collective well-being. A researcher should use multidisciplinarity to observe a phenomenon in its entirety and not only its alignment of thought, federations, committees, and knowledge; to get to understand it is necessary to exploit more tools and more disciplines. The article discusses the importance of the fluids (or liquid fascia) in maintaining the shape and function of the human body, as, currently, many texts forget how much body fluids are fundamental for understanding structural dynamics (bones and muscles, fibrils, and cells). By revisiting the current literature, the text wishes to highlight how the liquid fascia determines body adaptation in the presence of mechanical stress. Without fluids, there would be no body shape that we know.

## Introduction and background

The mechanical model involving the skeletal muscle system and the fascial continuum is known as biotensegrity. In the 1960s, the designer R. Buckminster Fuller conceived and put into practice the structural and architectural concept of tensegrity (tensional integrity): continuous tension with discontinuous compression [1]. He was inspired by a sculpture by the artist Snelson in 1948 [2]. This principle made it possible to build architectural projects that maintained their shape, despite external mechanical stresses or temporary changes in the external structure; the tensional balance allows the continuation of function and form [1]. In 1977, Dr. Robbie was the first to equate the concept of tensegrity in the biological field and, precisely, with the spine and with the musculoskeletal system, trying to understand the dynamics of the forces acting on the structure's living (biological), maintaining form and function [2]. According to his vision, the muscular and bone system could be seen as a system of biological tensegrity [2]. In the late 1970s and then in 1985, Ingber equated the concept of tensegrity with the cell, where microtubules manage the mechanical tensions (structures with continuous tension) produced by the actomyosin protein complex (structures with discontinuous compression) [2]. The ultimate goal is to conceptualize the phenomenon of mechanotransduction, that is, the ability to transmit mechanical information inside and outside the cell through the structures themselves that make up the cell and, at the same time, obtain the maximum possible adaptation [1-2]. In 1981, with an abstract at the 34th Annual Conference on Engineering in Medicine and Biology, Dr. Levin coined the term we universally know: biotensegrity [2]. The theoretical model of biotensegrity considers bones and muscle tissue (and connected) as the component of discontinuous tension and the component in pre-stress, respectively [2]. The philosophy of biotensegrity can be applied to the whole body, as well as to every single body area, up to the cell, as in a previous article, where I have shown in detail the different connections [3]. The model marries the definition of what is the fascial continuum, according to Fascia Nomenclature Committee: “The fascial system includes adipose tissue, adventitia, neurovascular sheaths, aponeuroses, deep and superficial fasciae, dermis, epineurium, joint capsules, ligaments, membranes, meninges, myofascial expansions, periosteum, retinacula, septa, tendons (including endotendon/peritendon/epitendon/paratendon), visceral fasciae, and all the intramuscular and intermuscular connective tissues, including endomysium/perimysium/epimysium” [4]. What is not taken into consideration when trying to understand how to correctly apply this model (mechanical model), is the function of body fluids [5]. Our vision of what should be considered a fascia also includes body fluids: “The fascia is any tissue that contains features capable of responding to mechanical stimuli. The fascial continuum is the result of the evolution of the perfect synergy among different tissues, liquids, and solids capable of supporting, dividing, penetrating, feeding, and connecting all the regions of the body, from the epidermis to the bone, involving all its functions and organic structures. This continuum constantly transmits and receives mechanometabolic information that can influence the shape and function of the entire body. These afferent/efferent impulses come from the fascia and the tissues that are not considered as part of the fascia in a biunivocal mode. In this definition, these tissues include: epidermis, dermis, fat, blood, lymph, blood and lymphatic vessels, tissue covering the nervous filaments (endoneurium, perineurium, epineurium), voluntary striated muscle fibers, and the tissue covering and permeating it (epimysium, perimysium, endomysium), ligaments, tendons, aponeurosis, cartilage, bones, meninges, and tongue” [4]. The biotensegrity model does not match the presence of body fluids, as it does not take them into consideration. Another model called fascintegrity and created by the Foundation of Osteopathic Research and Clinical Endorsement (FORCE) group involves blood and lymph (specialized connective tissue) [4]. The human body is made up of fluids, from its entirety to the individual cell; without the presence of fluids, the human body or the single-cell could not adapt, could not survive and would not have the shape we know [3]. In the 21st century, we are still debating whether the biotensegrity model is a valid model [6]. The model is valid when fluids are not taken into consideration. Dissecting the understanding of a phenomenon means observing a three-dimensionality of different interpenetrations of scientific concepts. Continuing to pursue a theoretical model inevitably leads to confusion; the confusion does not arise from the multidisciplinarity of thought but from the marmoreal intellectual fixity. A model is used to advance and not to stop. As a model, it is a starting point that should only be defended when its total validity is demonstrated; currently, there is no experimental scientific study that proves validity on the living. But this is precisely the richness of a model, that is, to stimulate research and the advancement of the understanding of what happens in the biological sphere. The fascintegrity model includes fluids in its philosophy but, like the previous model, it is a theoretical approach, which needs further study [3]. The theme of the review is to highlight the importance of fluids on the ability of the cell and tissues to function and maintain the shape during biological activities. The ultimate goal is to stimulate further growth in the vision of what is the body system or fascial continuum.

## Review

The human body contains different fluids or liquid fascia with different tasks; all fluids can come into contact with each other [7]. The same cannot be said for muscles or bones or solid fascia. We can recognize blood (arterial and venous), lymph, cellular fluids (intracellular, extracellular), and cerebrospinal fluid [8-11]. Fluids play an extraordinary role in maintaining bodily functions and, therefore, the shape. The movement or quantity of fluids implies constant adaptation as they generate mechanical force [12]. For example, every time the body changes posture, the blood system changes the space occupied, altering the pressures. The vestibule must send information to the nucleus of the solitary tract, which last receives baroreceptor information from the aortic arch and the carotid glomus, to better manage blood pressure and posture [13]. Blood pressures affect posture. Another example concerns breast cancer cells. AMP-activated protein kinase (AMPK) has the task of monitoring cellular energy status and plays a fundamental role in the behavior of cancer cells. Generally, it is believed that the physical microenvironment primarily influences the response of cancer cells, a recent in vitro study has shown the importance of the fluid footprint on the tumor cell response [14]. Researchers have shown that steroid receptor coactivator (Src) and focal adhesion kinase (FAK), which are non-receptor intracellular structures, are extremely sensitive to shear stress from intracellular fluids and are capable of influencing AMPK and tumor proliferation [14]. The flow of fluids generates energy. Some chemists have developed a tiny battery that works by extracting energy from body fluids. It can be placed in contact with body fluids containing glucose, under the skin or in the backbone. Batteries operate using biological fluids [15]. Body fluids emit light, in particular, the blood emits biophotons or ultra-weak photon emission (UPE) [16]. Light is an electromagnetic wave but it also acts like a stream of corpuscles. A study shows that the two natures of light, the corpuscular and the wave nature, can be detected simultaneously in the same physical system [17]. The release of biophotons has a pattern, a recognizable rhythm; this biological behavior is equivalent to a communication system, probably, for the nervous system and for other body systems [16]. Fluids can make sounds like phonons. Phonons (oscillatory waves) are "quasiparticles" that carry sound and heat and, once again, carry information from one atom to another [18-19]. UPE and phonons interact with the biological response of the organism, but yet, there are no studies involving these notions in the theoretical model of biotensegrity.

Fluids and embryogenesis

**Figure 1:**
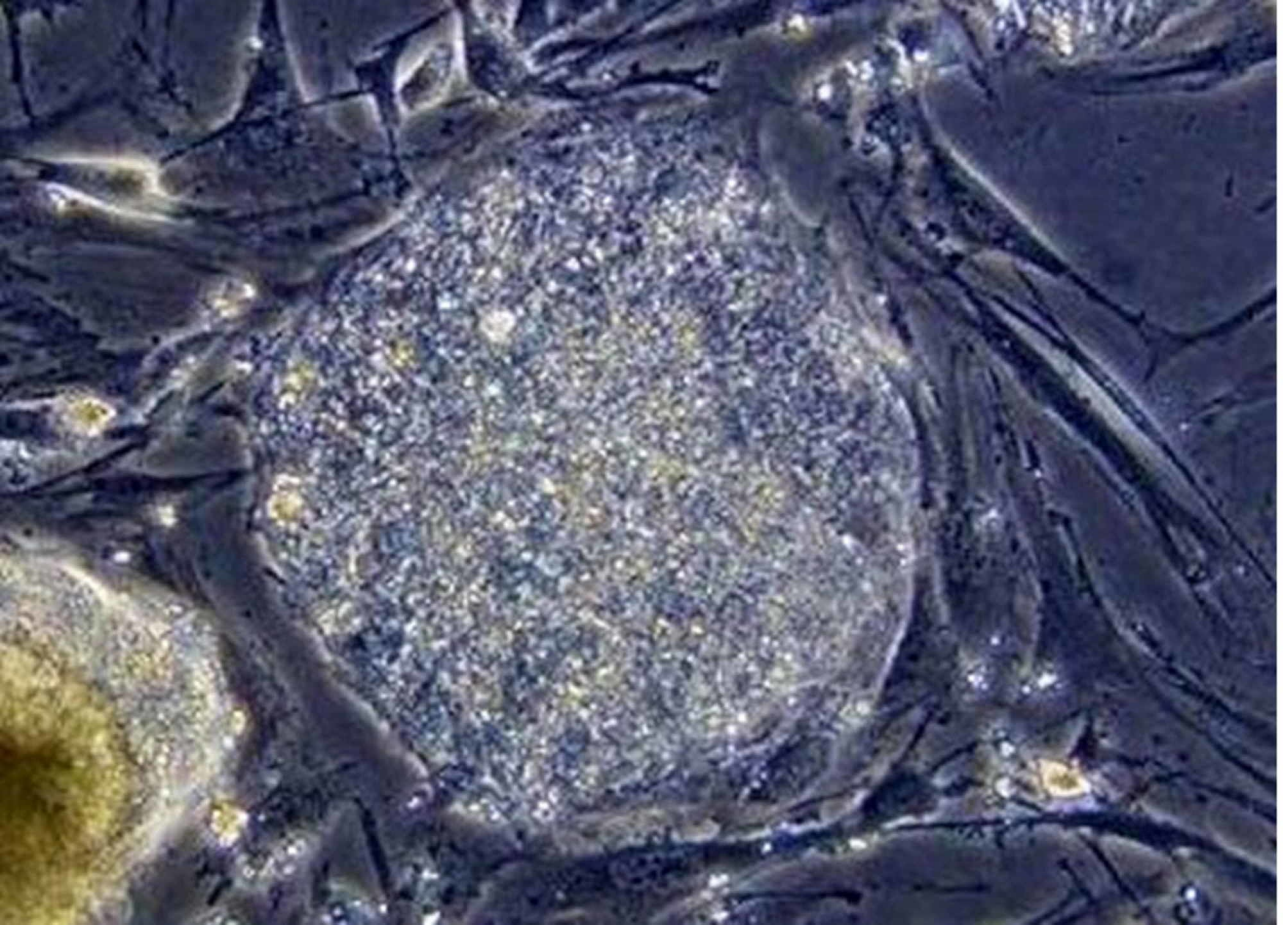
Stem cell taken from amniotic fluid; amnion is a precious container of stem cells. Three milliliters of amnio extracted during amniocentesis are able to supply 20,000 to 30,000 stem cells. The image is taken from the Don Gnocchi chemistry and research laboratory in Milan.

The structure adapts to fluids; the fluids with their movement, quantity, flow speed, and vector mode determine the shape of the tissues. The form and function of the fascial continuum are primarily given by fluids. Let us see some examples. The morphogenesis of the cardiac structure is based on the thrust of the fluids. The shear stress deriving from the flow of fluids specularly determines the shape of the cell, while the pressure of the fluids themselves determine the boundaries and functions of the cell itself: the fluids shape the form and function at the embryological level [20]. Another study confirms this concept, where researchers demonstrate that fluids and their behavior stimulate correct cell division during embryogenesis [21]. The precise size and symmetry of the organs, as well as their perfect position and function, depends on the fluids. The inner ear, an extraordinary precision organ, is morphogenetically stimulated by the quantity of fluids (hydrostatic pressure) present during embryogenesis; the same pressure manages the quantity of fluids that can intervene [22]. The fluids manage themselves through pressure feedback and, at the same time, influence the duration of growth and the size of the different tissues [22-23]. The presence of fluids influences the synthesis of chalones (mitotic inhibitors) [22]. The latter manages the duration of growth, such as somatostatin for skeletal muscle, bone type 3 morphogenetic protein (BMP3) for bone tissue, and protein growth differentiation factor 11 (GDF11) for the nervous system [22]. During embryogenesis, fluids also influence substances that manage the size of the tissue or organ, such as insulin, target of rapamycin (TOR) signalling pathways and by the Hippo protein kinase (Hpo) [22]. The accumulation of fluids or morphogenic gradient allows cells to synthesize these substances and create self-control of the growth, shape, and function of tissues and organs or organogenesis [22-23]. The structure, like the various components that make up the cell (proteins, microtubules, contact proteins, and more) respond to fluids (inside and outside the cell); without the constant work of fluids (blood, lymph, interstitial fluids, intra and extracellular fluids) there would be no form or function (Figure 1).

Fluids and mechanotransduction in the living being

**Figure 2:**
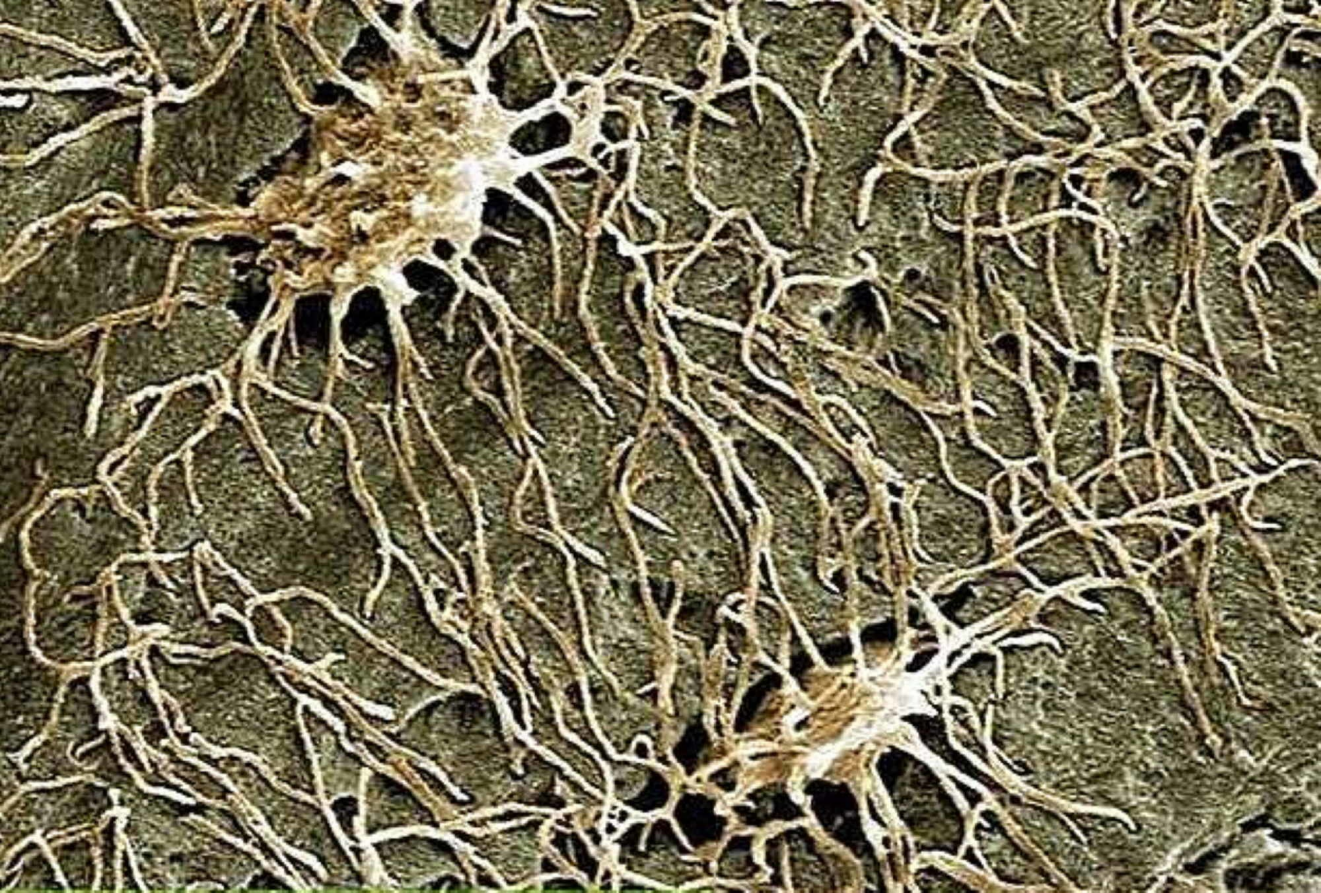
Electron microscope image highlights osteocytes. The image is taken from the Don Gnocchi chemistry and research laboratory in Milan.

Mechanotransduction is a fundamental mechanism for the adaptation of the human body, where, a mechanical stimulus alters the shape of the cell, which responds with an electrochemical cascade [24]. The classic chemical-mechanical model looks at calcium as the initiator of the contraction of the actomyosin complex, which complex is capable of deforming the cell [25]. The contraction is used to understand the surrounding mechanometabolic environment and to get the mechanical information to the nucleus of the cell; moreover, this deformation is felt by other cells [25]. The mechanical deformation of the cell is not only an adaptation but a means of communication [3]. This is biotensegrity. The means of spreading of this mechanical, electrical, and chemical information are fluids. The cell is full of cytoplasm, as well as the membrane that surrounds it is rich in fluids; externally we find the extracellular matrix with interstitial fluids and more consistent hydrostatic pressure changes due to the presence of vessels containing lymph and blood [3]. Without the fluids, the deformation of the cell would not occur, there would be no movement of the actomyosin complex, there would be no transport of biochemical or electrical activity [25]. The movement of cellular fluids occurs with self-regulating patterns (rhythmic waves); the waves produced not only carry the signals produced at distances and at higher speeds than the protein connections between the extracellular matrix and the nucleus, but the wave itself represents an electro-mechanical-chemical signal [25]. Unfortunately, we still lack many more detailed elements to understand this mechanism, but there is no doubt that this fluidic phenomenon is of primary importance. As written in a previous article: "The cell without the fluids does not move and cannot survive" [3]. The cell and the different tissues of the human body do not host fluids but are hosts of the fluids and carry their embryological fluidic imprinting. Fluids are important for the adaptation of an organ or contractile tissue in the embryogenic field. A study has shown how, during the contraction of muscle fibers, the resulting hypertrophic adaptation was allowed primarily not by the tension generated by the fibers but by the hydrostatic pressure of the interstitial fluids [26]. Interstitial fluids represent about 20% of body weight and, most likely, have their own pathways that have not yet been fully elucidated [27]. Pressure gradients are important for bone health, through penetrating fluids in bone tissue [28]. We can recognize many fluidic pathways, such as Volkmann and Haversian canals, the lacunar-canalicular system and the collagen-hydroxyapatite porosity, all of the size of microns [29]. The flow of fluids stimulates the proliferation and maturation of bone tissue, controlling the balance between osteoblasts and osteoclasts, and the synthesis of bone morphogenetic proteins (BMPs), osteopontin (OPN), and osteocalcin (OC) [29]. The bone tissue could not survive without the presence of this constant flow of fluids [29-30]. The flow can be laminar, rhythmic, constant, oscillatory, and bidirectional; the passage of the fluids activates the first passage of the mechanotransduction (mechanocoupling) and then the second passage (biochemical coupling), up to the chromaffin (figure 2) [29].

The organs also respond to the presence of fluids. The pressure of the fluids inside the kidneys influences the podocytes, involving cyclooxygenase-2 and prostaglandins type 2 in the mechanical response and other molecules (Akt-GSK3β-β-catenin) for correct adaptation (and shape) and kidney function [31]. The constant passage of fluids maintains the shape and function of the myocardium, stimulating the potassium channels (for correct electrical function), stimulating correct repair (through the intervention of stem cells) [32-34]. According to a model to understand the importance of mechanotransduction in the presence of fluids, the fluid mosaic model, highlights that the cell does not behave morphologically in unison during the passage of fluidic-mechanical information but reminds a lot of the fractal concept [35 ]. This happens because fluids are not rigid structures and do not intervene with their footprint equally on the cell surface or from inside [35]. In addition, the membrane itself can be rigid or soft, depending on the fluidic tension of the cell or the external environment [35].

Need for a new fascial model

**Figure 3:**
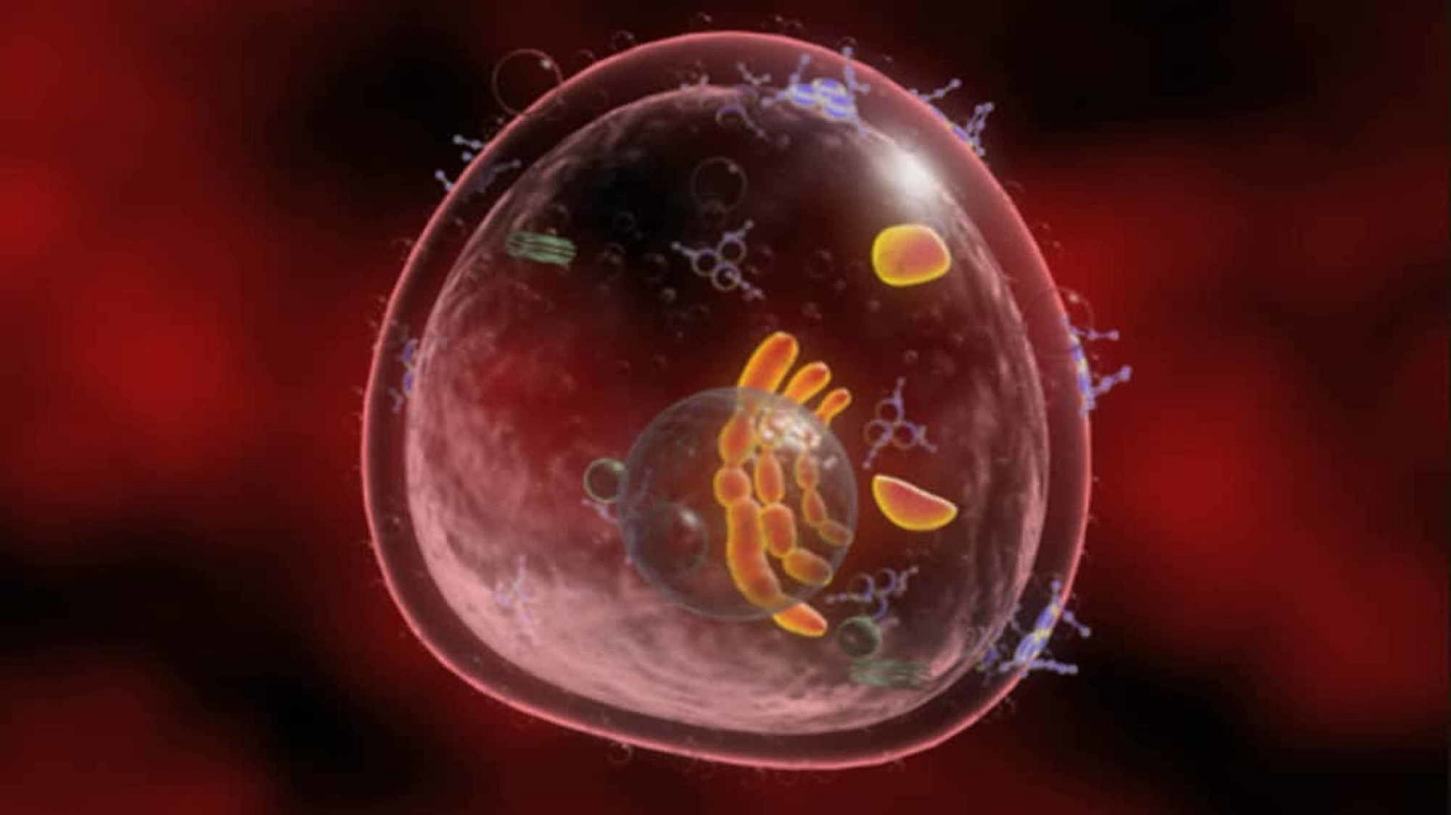
The figure shows the representation of a T lymphocyte, where the fluids are inside and outside the cell. The image (computer processed) is taken from the Don Gnocchi chemistry and research laboratory in Milan.

“It is rare that scientific models are not modified from their original forms to reflect new observations or data that were not anticipated when the models were proposed [35].” The presence of fluids weakens the concept of biotensegrity. Through tensegrity, scientists built equipment sent into space, but not through the theoretical model of biotensegrity [36]. The latter concerns the dynamics of the human body. We know that biotensegrity bases its foundations on the previous architectural model, with rigid and pre-stressed structures (bones and muscles); we know that the cell has been equated with this biological vision but, body fluids have not been taken into sufficient consideration [3]. We know that the presence of fluids is fundamental for the growth and survival of cells, as well as for the maintenance of the shape and function of tissues and organs [37-39]. In addition, the temperature itself within the different cellular fluid compartments is not homogeneous [40]. This means that the biological behavior of the cell and its extracellular relationships will vary greatly. The displacement of the cell depends on the fluids present and on the rheological nature of the same, as well as the transport capacity of the various biochemical substances [41-42]. In the theoretical conception of biotensegrity, the branch of physics that studies fluid dynamics has not been taken into consideration. Fluid dynamics studies the behavior of fluids in motion, in relation to the causes that determine it [43-44]. Understanding fluids will mean to understand how cells behave and how pathologies develop [44-45]. A new model proposed is called fascintegrity, in which the presence of body fluids is considered [3]. The latter is always a theoretical concept, where detailed analyzes are missing to apply this model in the living, but it is a step forward. How to integrate fluids into the fascial continuum? Fluids have many characteristics, such as viscosity, temperature, direction, turbulence, speed, rhythm, and elasticity [46-48]. The variables will determine health or pathology [49]. I would add, in a fascial view, that fluids have rigidity characteristics (a fluid is incompressible); they can create an environment of pre-stress (variation of hydrostatic pressure) and discontinuous tension (the oscillatory rhythm). Unlike the solid fascia, whose characteristics do not change, the liquid fascia has interchangeable peculiarities. The fascial continuum is a fluidic network where we find the solid fascia immersed into the fluidic fascia. Dr Hazzard wrote: “..a solid is rigid and retains its shape, whereas a liquid conforms to its surroundings. [50]”. I would add that fluids not only have the ability to adapt to the solid but also are the fluids that make the solid capable of adaptation (Figure 3).

## Conclusions

Mechanotransduction is a fundamental mechanism for the adaptation of the human body, where a mechanical stimulus alters the shape of the cell, which responds with an electrochemical cascade. The mechanical deformation of the cell is not only an adaptation but also is a means of communication. The mechanistic view of the human body as the theoretical concept of biotensegrity does not take into due consideration the presence and the fundamental role played by body fluids, blood, lymph, interstitial and intracellular fluids are the possibility of cell survival. Survival means having the ability to adapt. Body fluids or liquid fascia, as illustrated in the article, influence the shape and function of the cell and organs, making the solid fascia capable of adapting through a mechanical transduction.
